# How Can Nursing Teams Respond to Large-Scale COVID-19 Screening?

**DOI:** 10.3389/fpubh.2021.681255

**Published:** 2021-10-29

**Authors:** Huihan Zhao, Yu He, Fang Brister, Li Yang, Gaoye Li, Ying Ling, Yanping Ying

**Affiliations:** ^1^Department of Nursing, The First Affiliated Hospital of Guangxi Medical University, Nanning, China; ^2^Department of Clinical Laboratory, The First Affiliated Hospital of Guangxi Medical University, Nanning, China; ^3^Clinical Research Unit, UT Southwestern Medical Center, Dallas, TX, United States

**Keywords:** nurse, large-scale, COVID-19, nucleic acid screening, 10 in 1 test

## Abstract

The COVID-19 virus has devastated lives and economies worldwide. The responses of nursing teams to large-scale COVID-19 screening have rarely been addressed or described. The aim of this study is to introduce an efficient response strategy for nurses in large-scale COVID-19 screening. A new COVID-19 case was confirmed on Jan 14, 2021 in Nanning, China. Immediately, a large-scale COVID-19 screening was launched and ran from Jan 14 to Jan 17, 2021. Our nurse team responding to the screening included three major components: (1) establishing a leadership group and a nucleic acid sampling emergency team; (2) defining, conducting, and evaluating nurse training; (3) implementing efficient sampling schemes (10 in 1 mixed sample technique). A total of 500 nurse volunteers were recruited and divided into three echelons. A total of 353 trained nurses were sent to 65 sampling stand stations. In cooperation with nurses from other health institutions, samples were collected from a total of 854,215 people in only 4 days for 2019-nCOV nucleic acid screening. The preparation and efficient response strategies used to conduct this screening may provide a baseline reference for future large-scale COVID-19 screening worldwide.

## Introduction

Since late 2019, the unprecedented COVID-19 pandemic has devastated lives and economies worldwide. The Chinese government has been implementing stringent control measures in an attempt to fight the pandemic. While efforts to date have been successful at controlling COVID-19 transmission. However, small clusters in some provinces and cities remained at the beginning of 2021. There were no new confirmed cases in the past 328 days in Nanning, China until Jan 14, 2021 (1) when a fresh COVID-19 case was confirmed. Local government agencies, institutions, and communities responded rapidly; health institutions were especially active and involved. On the same day, an epidemiological investigation was conducted by the public health agency and the positive case's contact tracing on the 16 days prior to his diagnosis were presented, which involved 1,421 contacts including 321 close contacts (2). All contacts were then managed in accordance with strict containment requirements.

Based on the contact tracing, local authorities identified the key areas of potential spread. On Jan 15, 2021 a large-scale COVID-19 screening was launched and expanded outward in concentric circles from this key area to further expand the nucleic acid detection region and increase the detection volume. To cover this large area, many skilled and trained nurses would be urgently needed to collect the required samples. Sampling practices place medical personnel at high risk of contracting the virus from infected persons (3). Additionally, it was estimated that millions of people would agree to COVID-19 screening during these few days. Facing this challenging task, how did the nursing teams respond to the predicted large-scale COVID-19 screening efficiently?

## Preparation

The First Affiliated Hospital of Guangxi Medical University as a general hospital is the largest hospital in Nanning Guangxi with 2,750-beds and 2,092 registered nurses. Preparations were initiated as follows:

- A leadership team was established in the hospital with the clear duty of effectively and efficiently conducting and coordinating a large-scale screening process.
- The leadership team consisted of a comprehensive coordination group, supply support group, a staff support group, a hospital infection prevention and control group, a traffic support group, and a public communication group.
- The nursing department developed a detailed large-scale nucleic acid detection sampling scheme and cleared the main work content.
- A total of 500 volunteer nurses were recruited to formed a nucleic acid sampling emergency team in the preparatory phase.
- In order to ensure sufficient capacity to meet continuing needs, the nurses were divided into three echelons: a first echelon, a second echelon, and a reserve echelon.
- The training content for the nurses was developed that covered the sampling region, object, locus and situation, work flow, and COVID-19 disease knowledge as well as related practice training that focused on tertiary prevention for medical staff, hand hygiene, naso/oropharyngeal swab collection, and 10 in 1 (OR 5 in 1) mixed sampling technique.
- The various training methods included field practice, simulation training with multiple simulated emergency scenarios.
- Each team member was required to pass the final exam to ensure they had the knowledge and ability to complete the urgent task.

## Action

A total of 353 trained nurses were included in the large-scale screening and were responsible for 12 sampling sites consisting of 65 sampling stands. At least 2–3 trained nurses were set up at each sampling stand station for 4 h shifts. Nucleic acid detection sampling was conducted uninterrupted for 24 h. In cooperation with nurses from other health institutions in Nanning, samples were collected from a total of 854,215 people during a period of 3 days from Jan 14 to Jan 17, 2021. On Jan 18, 2021, Guangxi local health authorities reported that all the test results were negative (2).

## Discussion

Pharyngeal swab collection was a key step for the screening and affected the test result and thus the effectiveness of COVID-19 prevention and control. This collection was most often carried out by trained nurses since naso/oropharyngeal swab sampling required more skilled and trained nurses in order to produce a good sample and minimize false-negative results (3). Before the large-scale COVID-19 screening several organizational activities were necessary. Preparation for these activities was done in advance and included establishing the leadership group, identifying sampling schemes, and preparing nurse training curricula so that the program could be launched at any time. The nursing department identified sources of additional nurses and prepared to train them rapidly. Simulation training was important in case of unexpected event.

In order to improve the efficiency of nucleic acid detection, a new 10-in-one mixed sampling technique was applied, which differed from pooling samples and involved sampling naso/oropharyngeal swabs for 10 persons sequentially. Then, the swab heads were put into the same virus preservation tube during collection process, followed by enclosed transport, inactivation and testing (4). Any mixed samples with negative results were eliminated, and no further individual tests were conducted, while with mixed samples with positive results, further individual tests were conducted to determine who was infected with 2019-nCoV.

In China, the technique of the 10 in 1 mixed sample tests was recommended for large-scale 2019-nCoV nucleic acid screening by the Medical Treatment Group of the State Council in Response to Novel Coronavirus Pneumonia's Joint Prevention and Control Mechanism (4). The 10 in 1 test is a cost-, time-, and staff- saving approach. Obviously, individual sample tests have overburdened the limited resources of both staff and supplies. The mixed sample approach may reduce the number of samples submitted, thus reducing the test load during large-scale population screening, especially in the times of sudden and heavy inflow of test requests for pandemic surveillance. Sample pooling strategies have been shown to increase overall testing efficiency, with good specificity and only a slight loss of sensitivity (5). One study also suggested that pooling a maximum of 10 samples per pool can increase the diagnostic capacity of labs significantly without losing quality (6). Moreover, these sample pools are more effective in testing clinical samples in populations with low prevalence of SARS-CoV-2 (6). Compared to individual testing, the pooled-sample testing strategy requires around 76–93% fewer tests done in low to moderate prevalence settings (7). The 10-in-one mixed sampling (pool all samples together into the same container at the collection site) and pooling sample strategies (medias are mixed in the laboratory into pools) are different but have similar principles. Two researches have evaluated the approaches and found the former approach enables high efficiency in COVID-19 screening without loss of sensitivity for screening of a complete population (8, 9). Current researches mainly focused on and recommended different pool-testing strategies including two approaches of pooling samples and different pool sizes in large scale COVID-19 screening which have been used in some countries in Asia, Europe, North America, South America et al. (10). Different pooling strategy implemented in COVID-19 screening are based on the prevalence or rate of positivity in community. To the best of our knowledge, most of pooling test in other countries is that media are mixed in the laboratory. Descriptions of 10-in-one mixed sample testing are limited. More data are required to validate 10-in-one mixed sample testing. Our present will be value reference during the ongoing COVID-19 pandemic.

**Figure 1:**
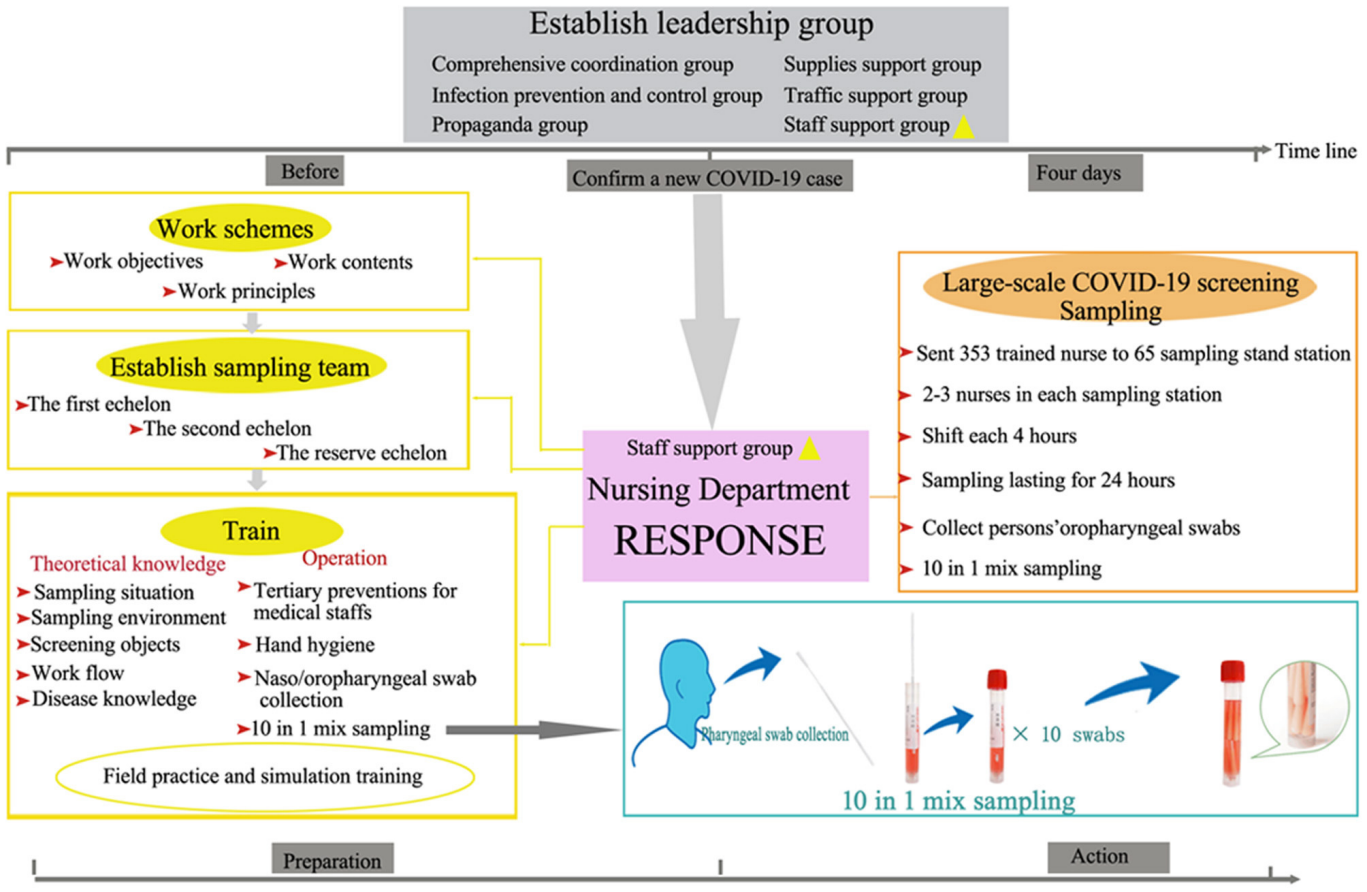
Nursing team response to large scale COVID-19 Screening. The response strategy included previous preparation and effective action. Each step was indispensable. The 10 in 1 mix samples technique which is different from pooling samples was applied in this process.

The Lancet Journal reported that the potential value of nurses was inestimably important for universal health coverage in 2019 (11). Nurses from across China were sent to Hubei province to fight the COVID-19 epidemic, performing well and displaying notable professionalism, and were praised at the beginning of 2020 (12). In addition, many nurses voluntarily joined this large-scale COVID-19 screening and their made significant contributions to the success of the program. The efficient response of the nursing team (Figure 1) played a vital role in the success of this screening. The large-scale nucleic acid detection sampling task that was successfully completed in 4 days confirms their assessment again. The response of China's nurses to this public health emergency has been rightfully recognized and praised by other healthcare professionals and the public. The potential value of nurses for global health has never been more apparent in the 18 months that COVID-19 has existed. Both public and private institutions should respect nurses and provide enough space for them to show their full value.

## Limitations

Although the response to this urgent large-scale screening was effective and efficient, it can still be improved. Three key limitations should be focused:

- Emergency medical team support should receive more attention so that any health-related accidents involving waiting persons, sampling nurses, associated healthcare personnel, and all on-site workers can be immediately addressed.
- Priority sampling stations should be set up and reserved for vulnerable persons including children, pregnant women, the disabled, and the elderly so that emergency situations due to long wait times can be minimized.
- Lastly, it would be better to add more security guards to maintain good order and social distance.

There is still much that must be done to defeat COVID-19 and nursing teams will continue to perform a critical role on the front lines of that battle. It is hoped that our brief presentation of the work done in Nanning, China will provide a starting point for further work on large-scale COVID-19 screenings worldwide.

## Contribution to the COVID-19 Prevention

We presented the nurse team response to the large-scale COVID-19 screening which included establishing leadership group, creating a nucleic acid sampling emergency team, developing sampling schemes, nurses training as well as efficient sampling action. Besides, we also present the 10 in 1 mixed sampling, which differs from pooled sampling. The 10 in 1 mixed sampling means sampling naso/oropharyngeal swabs for 10 persons sequentially and then the swab heads are put into the same virus preservation tube. The approach taken in this large-scale screening activity resulted in the testing of over 850,000 persons during a 4 days period. Similar screening activities, if conducted worldwide, could significantly reduce the COVID-19 pandemic's impact by rapidly focusing prevention activities on areas with higher infection levels.

## Data Availability Statement

The original contributions presented in the study are included in the article/supplementary material, further inquiries can be directed to the corresponding author/s.

## Author Contributions

HZ and YH: designed this study and participated in collecting data and data analysis, writing the manuscript, and contributed to picture processing and drafting. FB: participated in writing and revising the manuscript. YY and YL: revised and finalized the manuscript. LY and GL: assisted with the study design, collected the related information from website, and revised the manuscript. All authors contributed to the article and approved the submitted version.

## Funding

This work was partially supported by Climbing Project of Nursing Clinical Research (YYZS2020025 and YYZS2020031) and Self-Funded Plan Projects of Guangxi Health Commission (Z20190398).

## Conflict of Interest

The authors declare that the research was conducted in the absence of any commercial or financial relationships that could be construed as a potential conflict of interest.

## Publisher's Note

All claims expressed in this article are solely those of the authors and do not necessarily represent those of their affiliated organizations, or those of the publisher, the editors and the reviewers. Any product that may be evaluated in this article, or claim that may be made by its manufacturer, is not guaranteed or endorsed by the publisher.
